# Hidden Hybridization and Habitat Differentiation in a Mediterranean Macrophyte, the Euryhaline Genus *Ruppia*

**DOI:** 10.3389/fpls.2020.00830

**Published:** 2020-07-10

**Authors:** Lise Beirinckx, Bram Vanschoenwinkel, Ludwig Triest

**Affiliations:** ^1^Ecology and Biodiversity Research Group, Plant Biology and Nature Management, Vrije Universiteit Brussel (VUB), Brussels, Belgium; ^2^Community Ecology Laboratory, Department of Biology, Vrije Universiteit Brussel (VUB), Brussels, Belgium; ^3^Centre for Environmental Management, University of the Free State, Bloemfontein, South Africa

**Keywords:** *Ruppia*, hybridization, habitat differentiation, cryptic diversity, coastal wetlands, microsatellites

## Abstract

In many aquatic plant taxa, classification based on morphology has always been difficult. Molecular markers revealed that the complexity in several of these aquatic taxa could be addressed to recurrent hybridization events and cryptic species diversity. The submerged macrophyte genus *Ruppia* is one of these aquatic genera with a complex taxonomy due to the absence of clear distinguishable traits and several hybridization events. Two species co-exist throughout Europe, *R. maritima* and *R. spiralis* (previously known as *R. cirrhosa*), but recent molecular studies also found several indications of hybridization, introgression and chloroplast capture between these species. However, the full extent and frequency of hybridization and introgression in this genus has not been studied so far, nor is it clear how these hybrid lineages can co-exist locally with their parental species. In this paper, we wanted to detect whether a single coastal wetland where both species co-exist can act as a *Ruppia* hybrid zone. As a case study, we chose the Camargue, a Mediterranean coastal wetland that harbors a wide diversity in aquatic habitats, especially in terms of salinity and hydro-regime. We sampled several *Ruppia* populations within this wetland. To identify each sample and reconstruct the local genetic structure of the two parental species and their hybrids, we used both chloroplast and nuclear microsatellite markers. Afterward, we tested whether different species had different habitat preferences. Our results confirmed that *R. maritima* and *R. spiralis* are two strongly divergent species with different reproductive ecologies and different habitat preferences. This prevents frequent hybridization and consequently we could not detect any trace of a recent hybridization event. However, we found several populations of later-generation hybrids, including a population of *R. maritima x hybrid* backcrosses. The hybrid populations occupy a different habitat and are genetically distinct from their parental species, although they tend to be morphological similar to parental *R. maritima*. Although local hybridization and introgression in *Ruppia* is less frequent than we expected, the taxonomy of *Ruppia* is complicated due to ancient hybridizations and several back-crossings.

## Introduction

Natural hybridization is an important mechanism in plant evolution. Newly formed hybrids can have traits that allow them to colonize new niches that are not occupied by their parental species (Arnold, 1997). Different models and examples predict that hybrid lineages are more likely to persist if they can colonize new niches because this enhances reproductive isolation (Buerkle et al., 2000; Kagawa and Takimoto, 2018) and avoids competition with the parental species (Cruzan and Arnold, 1993). For instance, *Helianthus* species found in extreme habitats such as the desert floor or salt marshes have hybrid origins (Rieseberg et al., 2007). In willows, persistent hybrid populations can co-exist with their parental species because they occupy more extreme niches in terms of temperature, nutrients and soil pH (Gramlich et al., 2016). However, in the absence of strong reproductive isolation, hybridization can be followed by one or several back-crosses with one of the parental species. This process is called introgression and allows gene flow between different species. It can strongly disrupt classic phylogenies based on one or more genes (Baack and Rieseberg, 2007; Mallet et al., 2016). If there is no selection for these introgressed genes and hybridization is rare, the traces of hybridization in the nuclear genome decrease after several generations of back-crossings. Eventually, this can result in chloroplast capture where the chloroplast - which is maternally inherited- is an introgressed organelle that has a different origin than the nuclear DNA (Rieseberg and Soltis, 1991; Tsitrone et al., 2003; Chan and Levin, 2005). As a result, introgression and chloroplast capture can be important indications for ancient hybridization beyond F1 and further generation hybrids (Masembe et al., 2006; Martin and Jiggins, 2017). These different levels of hybridization make it difficult to detect hybrids based on morphology. Besides, although several hybrid lineages are known to have an intermediate phenotype compared to the parental species or have a mosaic phenotype that combines characteristics from both parents (Burke et al., 1998; Tovar-Sánchez and Oyama, 2004; Salamone et al., 2013; Rüegg et al., 2019), this is not a general rule (Rieseberg, 1995). Many hybrids tend to resemble only one of their parental species (e.g., Rieseberg, 1995; Kaplan et al., 2009; Les et al., 2010; Takahashi et al., 2015) or hybrids can resemble other hybrids, although they have different parental species (Zou et al., 2017). Molecular markers have been essential to identify hybrid lineages and detect evidence of hybridization and introgression. However, detection of hybrids remains difficult and they can still complicate species delimitation and taxonomy (Duminil and Di Michele, 2009; Twyford and Ennos, 2012; Mallet et al., 2016).

**FIGURE 1:**
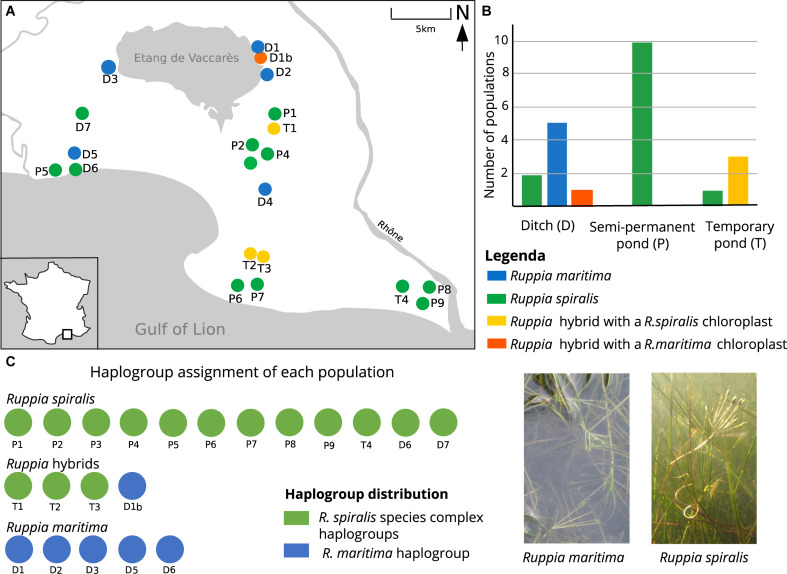
Locations and pond types of the sampled *Ruppia* populations in the Camargue and their haplotype distribution. Panel **(A)** maps the locations of the sampled *R. spiralis*, *R. maritima* and hybrid populations throughout the Camargue. Three types of habitats were distinguished: ditches (indicated with a D), semi-permanent ponds (indicated with a P) and temporary ponds (indicated with a T). Panel **(B)** shows how many populations of each *Ruppia* lineage were found in each pond type. Panel **(C)** shows the haplogroup assignment of each population. Only one population (D1b) of a hybrid origin was characterized with a *R. maritima* chloroplast (blue), three hybrid populations had a haplotype that could be placed in the *R. spiralis* species complex group (green). This *R. spiralis* species complex comprises several haplogroups that could not be distinguished based on fragment length. A picture of *R. maritima* and *R. spiralis* can be found in panel **(D)**: notice the long spiraling peduncle in *R. spiralis.* Hybrid population D1b can be considered as a subpopulation of pure *R. maritima* population D1: the were found as a mixed stand, within the same ditch. So far, population D1b is the first group of detected *Ruppia* hybrids with a *R. maritima* chloroplast.

The submerged macrophyte genus *Ruppia* is an example of a taxon in which hybridization, introgression and chloroplast capture have blurred taxonomy and species delimitation. The genus is distantly related to the seagrass family Posidoniaceae (Les et al., 1997) and well-known for its ability to cope with fluctuations in water-level and salinity (Verhoeven, 1979; den Hartog and Kuo, 2007). Taxonomy and identification within *Ruppia* have always been difficult because of the high morphological variability between populations and the absence of clear vegetative traits to distinguish different species (den Hartog and Kuo, 2007), a problem often encountered in aquatic plant taxa (Barrett et al., 1993). Throughout Europe, two *Ruppia* species are traditionally recognized, *Ruppia maritima* L. and *Ruppia cirrhosa* (Petagna) Grande (Reese, 1962; Verhoeven, 1979). However, recent scrutiny of the description of the lectotypification of *R. cirrhosa* concluded that this name has been used wrongly. Based on other herbarium specimens and species descriptions, *Ruppia spiralis* L. ex Dumortier is suggested as a more correct name, which will be used from hereon (Ito et al., 2017; den Hartog and Triest, 2020). The two species are most easily distinguished by their flower peduncle length. *R. spiralis* has long spiraling peduncles (>5 cm) which ensure the flowers to reach the water surface. This allows so-called epihydrophilous pollination: male pollen float over the water surface to the female flowers for fertilization. *R. maritima* has short peduncles (<5 cm) and the flowers remain submerged (Figure 1D). Their flower anatomy is expected to strongly promote self-fertilization (Verhoeven, 1979). Other diagnostic characteristics are the leaf tip shape and leaf width: *R. maritima* has small leaves (2–5 mm) that end in an acute leaf tip whereas *R. spiralis* leaves are broader (4–11 mm) and end obtusely (Verhoeven, 1979; Triest and Sierens, 2013; Mannino et al., 2015). However, these characteristics are more ambiguous, and identification remains difficult in the absence of flowers.

Molecular studies were performed to clarify the phylogenetic relationships within European *Ruppia* and to provide a barcoding tool to unambiguously identify *Ruppia* species. They confirmed that *R. maritima* and *R. spiralis* are two separate genetic entities that can be placed in different chloroplast haplogroups (Ito et al., 2010; Triest and Sierens, 2010), have different ITS- regions (Ito et al., 2013; Triest and Sierens, 2013) and amplify different sets of microsatellite markers (Triest et al., 2017). Combining these molecular studies with ecological data, *R. maritima* is considered as a diploid, predominantly selfing species with an annual life cycle and a high seed set (Triest and Sierens, 2015).

Tetraploid *R. spiralis* has both annual and perennial populations (Verhoeven, 1979; Gesti et al., 2005). This species can reproduce sexual through outcrossing or vegetative through underground rhizomes. The importance of either sexual or vegetative reproduction highly varies between populations. This is not only reflected in the big differences in clonality between populations but also in the large variation in number of flowers and seed production (Martínez-Garrido et al., 2016, 2017; Triest et al., 2018). Unambiguous identification has also led to the suggestion that *R. spiralis* and *R. maritima* might have different habitat preferences: *R. spiralis* is expected to prefer larger and more permanent waterbodies (Verhoeven, 1979; Triest and Sierens, 2013; Mannino et al., 2015; Martínez-Garrido et al., 2016) whereas *R. maritima* might prefers smaller and more ephemeral habitats (Verhoeven, 1979; Triest and Sierens, 2013). However, several authors also reported *R. maritima* in permanent waterbodies (Verhoeven, 1979; Mannino et al., 2015).

Furthermore, these molecular studies also found several indications of hybridization events. So far, three groups of hybrids have been detected. The first hybrid lineage has a unique haplotype, the so-called haplotype E, that is unrelated to the *R. maritima* haplotypes, but its nuclear DNA is related to *R. maritima*. Therefore, this lineage is thought to be the result of an ancient chloroplast capture event between a currently unknown and probably extinct taxon as the maternal parent and *R. maritima* as pollen donor (Ito et al., 2013; Triest and Sierens, 2015; Martínez-Garrido et al., 2016). The second group of hybrids is the outcome of a *R. spiralis x haplotype group E* hybridization event, eventually followed by backcrossings with haplotype group E (Ito et al., 2013; Martínez-Garrido et al., 2016). Both lineages were detected in the Mediterranean, a potential hotspot for *Ruppia* diversification (Triest and Sierens, 2014). The third hybrid lineage was detected at the Atlantic coast in France and is characterized with a *R. spiralis* haplogroup, but the nuclear DNA seems introgressed with *R. maritima.* This lineage probably originated from a hybridization event between *R. spiralis* as the maternal parent and *R. maritima* as a pollen donor, probably followed by several back-crossings with *R. maritima* as pollen donor. The latter two lineages are both characterized by *R. spiralis* haplotypes. Although none of the encountered hybrid populations were described in detail in terms of ecology or morphology, microsatellite data suggest that they can reproduce sexually through outcrossing (Triest and Sierens, 2015; Martínez-Garrido et al., 2016).

Hybrid zones are secondary contact zones between two species (Harrison and Larson, 2016) and provide ideal study areas to investigate the frequency and consequences of hybridization events (Barton and Hewitt, 1985). In a classic hybrid zone, both parental species are found, often at the edge of their range, together with their mutual F1 hybrids. If these F1-hybrids are fertile, later-generation hybrids and backcrosses to either parental species can be found as well. In the parental populations around the hybrid zone, some levels of introgression can often be detected. However, hybridization can occasionally result in new combinations of traits that allow these hybrids to outperform their parental species under particular ecological conditions. Mosaic landscapes offer a wide range of habitats and ecological niches. They can provide opportunities for hybrids to colonize their own niche outside their parent’s range without long distance dispersal. The colonization of a new niche promotes (partial) reproductive isolation which can be further enhanced by positive selection on these hybrids. Even in the presence of low gene flow with their parental species, the occupation of a new ecological niche promotes the persistence of these hybrid lineages in sympatry with their parental species (Arnold, 1997; Buerkle et al., 2000). Coastal wetland areas are characterized by a wide range of different pond types with different salinities and are hence considered as a mosaic landscape. They promote regional co-existence of *R. spiralis* and *R. maritima* within the same area (Verhoeven, 1975) and are potential *Ruppia* hybrid zones. Due to the large variety of habitats present, wetland areas might also provide opportunities for hybrid speciation (Arnold et al., 2012). So-far, most encountered *Ruppia* hybrid populations were detected unintentionally in large regional studies. As a result, the presence of *Ruppia* hybrid zones, and the co-occurrence of hybrid lineages and their parental species is poorly documented. In addition, it is not known to what extent *Ruppia* hybrids may have different niches compared to the parental species.

Our main goal was to detect different *Ruppia* species and possible hybrids that co-occur within a coastal wetland area. We used both chloroplast and nuclear microsatellite markers to reconstruct the genetic structure and local distribution of the different lineages and detect the origins of older or more recent hybrid lineages. Secondly, we explored potential differences in habitat use between the different lineages. We chose the Camargue as a study area, a Mediterranean coastal wetland area in the south of France that represents a potential hybrid zone. This wetland area contains a large variety of aquatic habitats that are affected by seasonal variation in salinity and water level and is known to harbor both *R. maritima* and *R. spiralis* populations (Verhoeven, 1975; Triest and Sierens, 2014, 2015). The detection of F1 hybrids or recent introgression events would confirm that coastal wetlands could act as a *Ruppia* hybrid zone. The presence of hybrids of an older origin, as well as differences in habitat preference between different lineages could indicate the occurrence of hybrid speciation.

## Materials and Methods

### Sampling Strategy and Habitat Types

*Ruppia* plants were sampled in May 2014 from twenty locations within the French regional park of the Camargue (Figure 1A). In each location, we collected thirty different ramets at 1.5 m intervals along a linear transect. Each location corresponded to a delineated waterbody, and hence to a potential different population. Two populations were found within the same ditch system (populations D5 and D6) but were separated by >200 m of bare soil. Four populations (T2, T3, T4, and D1) were found in ponds that were too small to make a linear transect of 50 m hence sampling distance was reduced to 0.5 m. In total, we collected 600 plants. Collected plant material was dried on silica gel.

During the field sampling, we already tried to give a species name to each population, based on their morphological features. We used flower peduncle length as the main discriminative characteristic: *R. maritima* has small peduncles (<5 cm), *R. spiralis* has long spiraling peduncles (>5 cm) (Figure 1D). If flowers were absent, we looked at leaf morphology. *R. maritima* has narrow leaves (2–5 mm) with an acute leaf tip, although these tips can become more obtuse-like in older leaves. *R. spiralis* has broader leaves (4–11 mm), and obtuse leaf tips (Verhoeven, 1979; Triest and Sierens, 2013). Some populations without flowers were still in an early developmental stage. In these seedlings, leaf tip was a more useful characteristic than leave width. However, in adult flowerless populations, we used a combination of leaf width and leaf tip shape for identification. We did not look at these characteristics when flowers were present. Based on the above criteria, each species could be easily addressed as either *R. spiralis* or *R. maritima* (Table 1).

**TABLE 1:** List of sampled populations with the population names (Pop), number of ramets sampled in each population (Ramets), number of multilocus genotypes (MLGs), clonality (R) calculated after clone correction with the formula *R* = (*G*−1)/(*R*−1), mean number of alleles per locus (N_A_), mean number of effective alleles per locus (N_*E*_), observed heterozygosity (H_OBS_), expected heterozygosity (H_EXP_), inbreeding coefficient (G_IS_), percentage of missing values after removing all failed amplifications (% missing values), the salinity measured at the moment of sampling (salinity) and the peduncle length of a flower if flowers were present.

Pop	Ramets	MLGs	R	N_A_	N_*E*_	H_OBS_	H_EXP_	G_IS_	Missing values (%)	Salinity (μs/cm)	Flower peduncle length
***R. spiralis***											
P1	29	29	1.00	5.23	2.59	0.61	0.54	−0.08	11.8%	24.4	>5 cm
P2	12	12	1.00	4.77	2.37	0.57	0.59	0.04	24.4%	39.6	Flowers absent
P3	30	30	1.00	6.58	3.13	0.65	0.65	0.00	12.6%	35.2	Flowers absent
P4	10	10	1.00	2.92	2.11	0.68	0.52	−0.25	9.3%	31.2	Flowers absent
P5	25	3	0.08	2.69	1.90	0.74	0.41	−0.81	14.6%	25.3	>5 cm
P6	28	28	1.00	5.46	2.82	0.64	0.57	−0.09	21.9%	57.4	>5 cm
P7	25	17	0.67	6.00	2.95	0.74	0.59	−0.24	23.4%	56.4	Flowers absent
P8	19	18	0.94	5.92	3.42	0.77	0.65	−0.20	8.6%	12.3	Flowers absent
P9	17	17	1.00	6.15	3.00	0.76	0.63	−0.21	13.9%	14.9	Flowers absent
D6	30	29	0.97	5.50	2.87	0.63	0.55	−0.17	12.7%	63.8	>5 cm
D7	29	28	0.96	6.75	3.17	0.64	0.58	−0.07	14.8%	62.1	Flowers absent
T4	8	8	1.00	4.00	2.60	0.71	0.58	−0.19	20.1%	69.4	>5 cm
***R. maritima***											
D1	16	2	0.06	1.10	1.03	0.00	0.02	1.00	3.1%	41.4	<5 cm
D2	13	1	0.00	1.01	1.00	0.00	0.00	/	3.1%	39.1	<5 cm
D3	25	2	0.04	1.10	1.04	0.00	0.03	1.00	3.2%	34.3	<5 cm
D4	15	2	0.07	1.10	1.05	0.00	0.03	1.00	11.3%	52.8	<5 cm
D5	21	1	0.00	1.00	1.00	0.00	0.00	/	2.9%	63.8	<5 cm
***Hybrids with R. spiralis markers***											
T1	26	14	0.52	2.23	1.60	0.43	−0.07	0.52	27.2%	16	<5 cm
T2	22	6	0.24	1.60	1.46	0.24	−0.22	0.24	36.5%	62.1	<5 cm
T3	22	22	1.00	3.30	1.81	0.34	0.04	1.00	51.5%	69.4	<5 cm
D1b	14	4	0.23	1.45	1.38	0.27	0.19	−0.42	30.1%	41.4	<5 cm
***Hybrids with R. maritima markers***											
T1	26	26	1.00	4.67	2.69	0.40	0.53	0.25	52.1%	16	<5 cm
T2	22	19	0.86	3.50	1.77	0.12	0.39	0.69	32.5%	62.1	<5 cm
T3	22	13	0.57	1.60	1.20	0.15	0.12	−0.22	2.7%	69.4	<5 cm
D1b	14	12	0.84	3.10	1.75	0.23	0.35	0.36	30,1%	41.4	<5 cm

The locations of the *Ruppia* populations could be categorized in three major habitat types: semi-permanent ponds, shallow temporary ponds and temporary ditches, which we, respectively, abbreviated in the population names as P, T, and D (Table 1). The semi-permanent ponds represented mainly large open waterbodies. Although pond size varies seasonally, all semi-permanent ponds had a surface area of more than 100 m^2^ during the sampling period. Depth during sampling was highly variable, ranging between 30 and 110 cm. Although the shallow outer margins of these ponds can dry out during summer, they only occasionally desiccate completely. Most of them are part of the large Vaccarès pond system or are abandoned salt pans. The second pond type, the temporary ponds, were shallow depressions in the dunes or salt marshes. These depressions are filled with rainwater during autumn and winter, but gradually dry during spring and summer. By the end of June, they are completely dry, leaving a cracked soil behind. This habitat type is particularly prone to yearly variation in temperature and rainfall (Verhoeven, 1975). During sampling, all temporary ponds were smaller than 100 m^2^, less than one meter deep and already reaching the end of their flooded season. The temporary ditches are the third pond type. They are characterized by steep sides and are clearly delineated. During sampling, water depth was more than one meter. The ditches are temporary habitats, but their hydro-regime is unpredictable because several of these ditches are regulated by sluices or used for agricultural wastewater. They have a typical linear shape but estimating the surface of these waterbodies is difficult because they are often part of a large and interconnected ditch system. Using the abovementioned criteria, all locations could be easily categorized in one of these three habitat categories.

### DNA Extraction and Marker Selection

Plant DNA was extracted from dried leaf tissue using the E.Z.N.A (R) HP Plant DNA Mini Kit Protocols (Omega Bio-Tek, Norcross, GA, United States). To genotype each specimen, we used a set of species-specific nuclear microsatellite markers that were combined in a multiplex reaction as well as a set of four chloroplast DNA markers. We used a QIAGEN multiplex PCR Plus kit to generate DNA-fragments with a PCR reaction in a thermal cycler (MJ research PTC-200 and Bio-Rad My Cycler). These fragments were run on an ABI3730XL sequencer (Macrogen, Seoul, South Korea). We manually scored the results with GeneMarker V2.20 (SoftGenetics LLC^®^).

**FIGURE 2:**
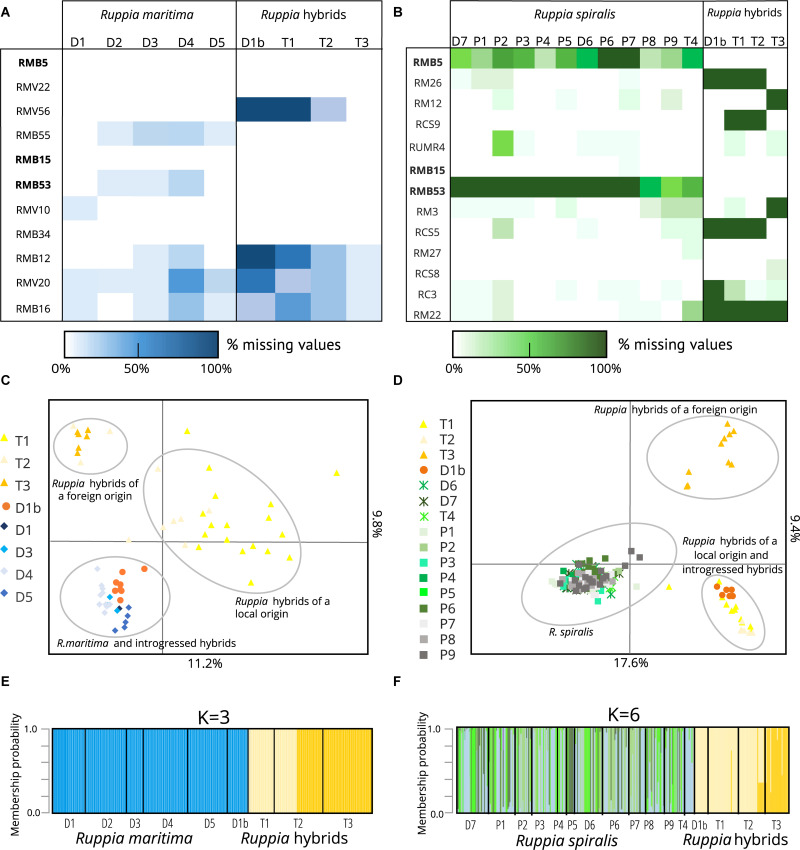
Overview of parental *Ruppia* lineages and two distinct groups of hybrids detected using two sets of microsatellite markers in the Camargue area. It is important to note that parental species only amplified using markers designed for that particular species while hybrids were amplified using both primer sets. Panels **(A,B)** show the amplification success of different lineages with the *R. maritima* specific markers and the *R. spiralis* specific markers, respectively. The markers in bold are the three assumed cross-amplifying markers, and where used in both sets of markers. In panels **(C,D)**, we see the clustering of different genetic lineages based on the alleles detected using both marker sets in a principal component analysis. Finally, panels **(E,F)** show the results of a complementary DAPC. This analysis shows the assignment of different genotyped individuals to clusters defined by the method. The principal component analysis **(C,D)** and the discriminant analysis of principal components **(E,F)** confirm the strong genetic differentiation between the hybrid populations and the pure species as well as the presence of two hybrid clusters. Based on the alleles present in the parental species, one group is expected to have a local origin, the other is presumably foreign. The eight samples of population D1b turn out to be backcrosses between the hybrids and *R. maritima:* they cannot be distinguished from pure *R. maritima* with the *R. maritima* markers, but group together with the hybrids when we used the *R. spiralis* markers.

Previous work on *Ruppia* revealed that very few microsatellites can cross-amplify in both species (Triest et al., 2017). Therefore, we genotyped each sample with a set of species-specific microsatellite markers that corresponded with their field-identification. Populations that we considered as *R. maritima* were genotyped with a set of eleven nuclear microsatellite markers that were originally developed on this specie*s* (Triest and Sierens, 2015). Assumed *R. spiralis* populations were genotyped with a set of fifteen microsatellites. This species-specific set comprised eleven microsatellite markers that were designed on *R. spiralis* plant material (Triest et al., 2017), as well as microsatellite marker RUMR4 that was originally designed on Chinese *Ruppia sinensis* plant material (Yu et al., 2009). The *R. spiralis* marker set also included three markers from the *R. maritima* multiplex (RMB5, RMB53, and RMB15) that are known to amplify well in several but not all *R. spiralis* populations (Triest et al., 2017). All populations had a variable number of samples with bad amplifications, which resulted in missing data. To visualize the amplification success of different markers (Figures 2A,B), both on population and individual level, we used R-package Poppr (Kamvar et al., 2014, 2015; R Development Core Team, 2018). We removed all samples where more than 50% of the markers had a poor or failed amplification result.

Because previous studies have already shown that morphological identifications of *Ruppia* are not always reliable (Ito et al., 2010; Triest and Sierens, 2013; Martínez-Garrido et al., 2016), we complemented each microsatellite genotype with a cpDNA haplotype. Using the sequences of four cpDNA markers [ccmp2, ccmp3, ccmp10 (Weising and Gardner, 2002) and TrnH-psbA (Kress and Erickson, 2007)], Triest and Sierens (2014) could distinguish various haplogroups within European *Ruppia* populations. Haplogroup D corresponds to *R. maritima* and is only far related to the remaining haplogroups (A, B, C, and E), that could be placed in a *R. spiralis* species complex. Although we considered amplicon length from a fragment analysis of these four cpDNA markers instead of their sequences, we should be able to distinguish at least both major species complexes (Triest and Sierens, 2014).

### Hybrid Detection

Usually, hybrids are detected with several microsatellite markers that have diagnostic alleles in both parental species (Buerkle, 2005). However, good cross-amplifying markers are lacking in *Ruppia* (Triest et al., 2017). As a consequence, we could not compare these two species and their putative hybrids in the same analysis and had to search for different methods to detect hybrids. As starting point, we had the morphological species determination for each sample, the species determination based on the chloroplast markers and the nuclear genotype based on one set of species-specific markers. If we assumed that a population had a hybrid origin, we amplified these samples also with the other species-specific nuclear microsatellite set.

First, we examined whether the species name we assigned to a population based on the morphological identification corresponded with the cpDNA identification. If there was a mismatch, we also amplified these populations with the microsatellite set that corresponded with the cpDNA. This allowed us to detect chloroplast capture or strong introgression and corrected for inaccurate morphological identifications.

Secondly, we looked for diagnostic alleles in the markers RUMR4 and RM3, both part of the *R. spiralis* multiplex. These markers were previously extensively tested on *R. spiralis* and *R. maritima* samples from all over Europe, including samples from the Camargue (Triest and Sierens, 2015; Triest et al., 2017). Both RM3 and RUMR4 had good amplification results in both species and alleles differed, respectively, at least seven and ten base pairs between *R. spiralis* and *R. maritima*. However, despite their diagnostic capabilities, these markers were not included in the *R. maritima* marker set because they are monomorphic in this species (Triest et al., 2017). In cross-amplifying markers, it is always uncertain to what extent these markers are able to describe the true genetic variation in both parental species, hence markers with little variation in one parental species should be handled with care (Thielsch et al., 2012). Therefore, we preferred to include mainly markers that could detect at least some variation in the *R. maritima* multiplex, especially when we take into account how little variation was previously found in this species (Triest and Sierens, 2015). Hybrids of a recent origin are hypothesized to have both diagnostic alleles.

Thirdly, we checked the three nuclear markers that were used in both microsatellite sets (RMB5, RMB53, and RMB15). Although these markers are known to amplify in both species, the amplification success in *R. spiralis* is highly variable across populations and they have a strong overlap in alleles between the two species (Triest et al., 2017). Therefore, the amplification success of these markers in *R. spiralis* populations could rather be a signal of an introgression event instead of a shared ancestral locus. If amplification is successful in both species, the distribution of alleles could provide information about the origin of a hybrid.

To visualize the population structure and the differences in microsatellite signature between either pure species or hybrid lineages, we constructed two Principal Component Analyses (PCA). We used R-package Adegenet for this analysis (Jombart, 2008; Jombart and Ahmed, 2011; R Development Core Team, 2018). The first PCA contains all samples that are genotyped with the *R. maritima* marker set (i.e., pure *R. maritima* and hybrid populations), the second PCA comprises all samples that are visualized with the *R. spiralis* markers (i.e., pure *R. spiralis* and hybrid populations) (Figures 2C,D). To check for the hidden population structure and clustering, we complemented both PCA’s with a Discriminant Analysis of Principal Components (DAPC) for each set of microsatellites (Figures 2E,F). DAPC is also implemented in the Adegenet package and uses K-means as a clustering algorithm to infer the genetic structure. It can work with polyploid data and does not require that populations are in Hardy-Weinberg equilibrium. We used the function “compoplot” to generate a graph that addresses the membership probability of each sample to the detected clusters (Jombart et al., 2010). Genetic structure of the different lineages.

Based on the previous species assignment, we considered three different datasets: a pure *R. maritima* dataset, a pure *R. spiralis* dataset and a *Ruppia* hybrid dataset. For population genetic analyses, we used the software Genodive, which can work with both diploid and polyploid data (Meirmans and Van Tienderen, 2004). To assign clones, GenoDive can account for scoring errors and missing values by setting up a genetic distance threshold below which similar multilocus genotypes (MLGs) are considered as identical multilocus lineages (MLLs). Setting up the threshold, we considered a stepwise mutation model. For *R. maritima* and *R. spiralis*, we chose to ignore the missing values; in the hybrid populations, we considered them as one mutation step. GenoDive can also calculate the probability that the observed clonal structure is the result of clonal growth rather than sexual reproduction (Gómez and Carvalho, 2000). If the calculated *p*-value was smaller than 0.05, we decided that the observed genetic structure was rather the result of sexual reproduction and all samples were kept in the population. If identical MLGs were the result of clonal growth, we kept only one representative sample in the population. After this clone-correction, we calculated the genotypic richness via *R* = (*G*−1)/(*N*−1) in which G is the number of different genets and N the total number of individuals (Arnaud-haond et al., 2007; Table 1).

To estimate the ploidy of a population, we counted the number of different alleles for each marker. If we detected three or four alleles for at least one locus in a sample, we considered that sample as tetraploid. Because triploids are mostly sterile, we don’t consider the possibility of a triploids in an outcrossing population. Besides, triploids were only rarely detected in *Ruppia* and never encountered in European *Ruppia* (Ito et al., 2010). Furthermore, if several samples in a population have three or four alleles for a single locus, we assume that the entire population is tetraploid.

Because most population statistics are developed for diploid populations, there are several restrictions for polyploids and comparisons among different ploidy levels. Above all, there is the problem of calculating observed heterozygosity: diploids have only one state of heterozygosity (e.g., AB) whereas tetraploids have partial and full heterozygotes (e.g., respectively, AABB and ABCD). Secondly, there is the problem of unknown allelic dosages in heterozygotes: if a sample has two different alleles for a single locus, e.g., A and B, it is difficult to deduct whether the true genotype is either AAAB, AABB or ABBB. This uncertainty strongly affects all allele-frequency based calculations (Dufresne et al., 2014). The software GenoDive (Meirmans and Van Tienderen, 2004) circumvents these two problems because it uses the principle of gametic heterozygosity to calculate the observed heterozygosity, which looks at the heterozygosity of the possible diploid gametes that can be drawn from a certain population (Moody et al., 1993; Meirmans et al., 2018). To counteract the problem of the unknown allelic dosages, GenoDive fills in the unknown alleles based on the observed allele frequencies in a population, following a maximum likelihood approach (De Silva et al., 2005). These corrections allow for a more correct estimation of the allele frequencies (Supplementary Material S1) and the frequency-based parameters, such as the inbreeding coefficient G_IS_ and fixation coefficient G_ST_ (Nei, 1973). However, the comparison of individuals and populations with different ploidy levels remains difficult because their allele frequencies respond differently to processes such as migration or drift. Ronfort et al. (1998) developed ρ, a statistic that measures population differentiation, but is independent of ploidy-level, rate of self-fertilization and typical polyploid problems such as rate of double reduction or polysomic inheritance (Meirmans and Van Tienderen, 2013). Parameter ρ can be calculated with GenoDive, using an AMOVA approach (Meirmans and Liu, 2018). Nevertheless, ρ is still based on observed and expected heterozygosity, which remain tricky concepts in polyploids. D_est_ (Jost, 2008) is another statistic to measure population differentiation that is unaffected by ploidy but is calculated based on the effective number of alleles. It is also independent of population size, but is highly affected by migration and mutation rates, and hence not well suited to describe population demographics (Ryman and Leimar, 2009; Meirmans and Hedrick, 2011). Parameters ρ and D_est_ were both calculated for each locus, and for population pairwise distances (Table 2).

**TABLE 2:** Parameters measured for each locus.

	*Ruppia spiralis*									*Ruppia hybrids*								
	N_A_	N_*E*_	% miss	H_OBS_	H_EXP_	G_IS_	G_ST_	ρ	D_EST_	N_A_	N_*E*_	% miss	H_OBS_	H_EXP_	G_IS_	G_ST_	ρ	D_EST_
RMB5°	10	1.8	68.8%	0.21	0.48	0.72	0.25	0.06	0.53	3	1.5	15.4%	0.00	0.55	1.00	0.39	0.46	0.57
RM26	14	4.3	3.0%	0.70	0.80	0.14	0.10	0.06	0.43	6	1.5	80.8%	0.50	0.80	0.01	0.34	0.35	1.00
RM12	9	2.7	3.3%	0.89	0.78	−0.30	0.02	0.08	0.05	3	2.0	33.7%	1.00	0.51	−0.98	−0.01	0.83	−0.02
RCS9	8	2.5	0.7%	0.75	0.61	−0.19	0.10	0.04	0.21	3	1.5	67.3%	1.00	0.69	−1.0	0.41	0.88	0.72
RUMR4	3	1.8	5.5%	0.79	0.62	−0.70	0.09	0.14	0.08	6	1.3	21.2%	0.26	0.60	−0.15	0.64	0.65	0.64
RMB15°	11	2.8	1.1%	0.72	0.64	−0.07	0.10	0.12	0.23	6	1.3	11.5%	0.06	0.60	0.75	0.59	0.64	0.63
RMB53°	3	1.3	93.7%	0.35	0.23	−0.54	/	/	/	2	1.0	1.9%	0.00	0.04	1.00	0.04	0.25	0.01
RM3	9	1.7	7.0%	0.55	0.52	−0.34	0.115	0.20	0.13	3	1.0	36.5%	0.00	0.46	1.00	0.95	0.78	0.01
RCS5	8	2.6	4.1%	0.70	0.72	−0.10	0.11	0.11	0.26	12	1.5	76.0%	1.00	0.87	−0.10	0.41	0.32	0.98
RM27	7	2.0	0.7%	0.99	0.52	−0.90	−0.02	0.01	−0.04	7	1.7	14.4%	0.74	0.67	−0.70	0.36	0.77	0.56
RCS8	8	1.9	1.5%	0.62	0.50	−0.22	0.13	0.09	0.16	6	1.3	22.1%	0.04	0.61	0.72	0.67	0.67	0.81
RC3	17	3.0	4.8%	0.79	0.76	−0.10	0.17	0.17	0.48	7	1.4	42.3%	0.50	0.77	−0.58	0.58	0.50	0.89
RM22	17	2.2	4.4%	0.59	0.57	0.04	0.10	0.10	0.23	/	/	/	/	/	/	/	/	/
Total	11.8	2.5	10.1%	0.66	0.59	−0.18	0.11	0.08	0.21	5	1.4	32.5%	0.40	0.50	−0.13	0.48	0.45	0.54

	***Ruppia maritima***									***Ruppia* hybrids**								

RMV22	2	1.1	3.4%	0.00	0.50	1.00	0.71	0.79	0.50	4	1.6	1.3%	0.29	0.46	0.19	0.63	0.61	0.43
RMV56	1	1.0	0.0%	0.00	0.00	/	/	/	/	4	1.2	41.0%	0.17	0.60	0.33	0.69	0.52	0.71
RMB5°	2	1.1	1.1%	0.00	0.18	1.00	0.48	0.45	0.11	3	1.1	1.3%	0.00	0.55	1.00	0.80	0.45	0.64
RMB53°	1	1.0	0.0%	0.00	0.00	/	/	/	/	2	1.0	0.0%	0.00	0.04	1.00	0.03	0.25	0.01
RMB55	1	1.0	9.1%	0.00	0.00	/	/	/	/	13	1.6	0.0%	0.37	0.71	0.15	0.59	0.72	0.62
RMB15°	1	1.0	0.0%	0.00	0.00	/	/	/	/	6	1.2	0.0%	0.11	0.44	0.77	0.59	0.64	0.63
RMV10	1	1.0	0.0%	0.00	0.00	/	/	/	/	7	1.9	0.0%	0.32	0.60	0.41	0.23	0.42	0.20
RMB34	2	1.0	2.3%	0.00	0.00	1.00	1.00	0.97	0.60	5	1.8	26.9%	0.41	0.65	0.13	0.20	0.15	0.43
RMB12	1	1.0	3.4%	0.00	0.48	/	/	/	/	7	1.7	37.2%	0.06	0.80	0.87	0.53	0.34	0.86
RMV20	1	1.0	13.6%	0.00	0.00	/	/	/	/	8	1.3	3.8%	0.23	0.42	0.27	0.38	0.33	0.23
RMB16	1	1.0	9.1%	0.00	0.00	/	/	/	/	5	1.3	24.4%	0.24	0.42	0.37	0.50	0.63	0.35
Total	1.3	1.0	5.4%	0.00	0.12	1.00	0.80	0.82	0.41	6.2	1.5	23.3%	0.22	0.58	0.39	0.55	0.47	0.50

*Loci that are present in both sets of markers are indicated with°. N_*A*_, number of alleles; N_*E*_, number of effective alleles; % miss, percentage of failed amplifications; H_*OBS*_, observed heterozygosity; H_*EXP*_, expected heterozygosity; G_*IS*_, inbreeding coefficient; G_*ST*_, fixation index; ρ, an index of population differentiation that is independent of the ploidy level and the rate of self-fertilization; D_*EST*_, another index of population differentiation, also independent of ploidy level, but it is calculated based on effective alleles instead of observed and expected heterozygosity.*

### Differences in Habitat Use?

Finally, we tested for an association between genetic lineage and habitat type with a two-sided Fisher exact test in R (R Development Core Team, 2018). We considered the three genetic entities, *R. spiralis, R. maritima* and the hybrids, separately and constructed a 3 × 2 contingency table for each showing the number of presences and absences in each of the habitat types. Pond type was considered as a three-leveled categorical variable (semi-permanent pond, temporary pond and ditch) and presence or absence of the genetic entity as a categorical variable with two levels. We counted the number of populations for each pond type where this group was, respectively, present or absent (Figure 1C).

## Results

### Detection of the Hybrids

Based on the morphological identifications, we considered eight populations as *R. maritima* and twelve as *R. spiralis*. Each assumed *R. maritima* population had many flowering ramets with short peduncles. Out of the twelve assumed *R. spiralis* populations, five had several flowers with long spiraling peduncles, seven populations were identified as *R. spiralis* based on leaf morphology (Table 1). When these results were compared with the chloroplast haplotypes, we found that three assumed *R. maritima* populations (T1, T2, and T3) had a chloroplast that could be placed in a *R*. *spiralis* species complex haplogroup. Using amplicon length of the chloroplast markers, we could clearly distinguish both species complexes, however this approach did not visualize all minor variants with certainty such as obtained from sequences.

The three populations with a mismatch between the morphological identification and the cpDNA haplogroup (T1, T2, and T3) were amplified with both sets of microsatellite markers. However, the *R. maritima* specific microsatellite markers had acceptable amplification success in these three populations, the observed amplification patterns strongly differ from pure *R. maritima*. While pure *R. maritima* had a very low genetic diversity (*N*_A_ = 1.3) and no heterozygotes (*N*_A_ = 1.3; *H*_OBS_ = 0.00; see Table 1 and section “Population Genetic Structure of *R. maritima* and *R. spiralis*”), populations T1, T2,‘ and T3 had a much higher allelic diversity (*N*_A_ = 5.8) and at least some levels of observed heterozygosity (*H*_OBS_ = 0.22). Besides, markers that had overall lower amplification success in the pure *R. maritima* populations (markers RMB12, RMV20, and RMB16), had even poorer amplification success in these three populations (Figure 2B). Marker RMV56 completely failed to amplify in population T1. These deviating patterns are also reflected in the PCA and DAPC based on the *R. maritima* microsatellite results (Figures 2D,F): populations T1, T2, and T3 are clearly separated from the pure *R. maritima* populations. This supports the presumption of a potential hybrid origin for populations T1, T2, and T3.

A comparable amplification pattern is found in fourteen samples of population D1. This population is identified as *R. maritima* and all samples have a *R. maritima* chloroplast. However, these fourteen samples completely failed to amplify two microsatellite markers (RMV56 and RMB12), had several unique alleles for the remaining seven markers (*N*_A_ = 3.1) and were the only *R. maritima* samples with some levels of observed heterozygosity (Table 1). All unique alleles discovered in these fourteen samples were re-encountered in assumed hybrid populations T1, T2, and T3. Therefore, we placed these samples in a subpopulation D1b and considered a potential hybrid origin for this subpopulation. Subsequently, we amplified them with the *R. spiralis* microsatellite markers.

To detect hybrids with the *R. spiralis* marker set, we first searched for diagnostic alleles with markers RUMR4 and RM3. All populations that were originally identified as *R. spiralis* had only *R. spiralis* diagnostic alleles (RUMR4: 124 and 126 bp; RM3: 231 and 233 bp). The assumed hybrid populations T1 and T2 were characterized exclusively by *R. maritima* diagnostic alleles (RUMR4: 136 bp; RM3:221 bp). Within population T3, marker RM3 did not amplify and marker RUMR4 was characterized by a unique allele of 131 bp which was not observed before in any other *Ruppia* population (Triest et al., 2017). In population D1b, marker RUMR4 was always heterozygous, with both *R. spiralis* and *R. maritima* alleles in each sample. Marker RM3 only detected *R. maritima* alleles in population D1b. For completeness, we must add that three *R. spiralis* samples were characterized with a new RUMR4 allele (128 bp; two samples in population P1, one sample in population D6). Marker RM3 also detected four new alleles *R. spiralis* (123, 124, 127, and 129 bp) but none of them was encountered in T1, T2, and T3.

Next, we considered the three cross-amplifying markers that were included in both marker sets. Markers RMB5 and RMB53 had very bad amplifications in populations with a *R. spiralis* chloroplast, except for populations T1, T2, and T3 (Figure 1B). Furthermore, they had high amplification success in all populations with a *R. maritima* chloroplast, including population D1b (Figure 1A). Marker RMB15 amplified well in all populations, but we observed differences in the number of alleles between the different *Ruppia* lineages. *R. spiralis* contained eleven different alleles, ranging from 161 bp until 181 bp. In *R. maritima*, this marker detected only one allele (167 bp). In population D1b, 167 bp was also the most dominant allele (allele frequency = 70.0%) out of four detected alleles (alleles 165, 169, and 175 bp). The assumed hybrid populations T1, T2, and T3 (*R. spiralis* chloroplast) are almost completely monomorphic for allele 173 (97%) and entirely homozygous for this marker.

Finally, microsatellite amplification success was highly variable between different markers and different populations of assumed hybrids, especially in the *R. spiralis* microsatellites. Within the *R. spiralis* specific set of markers, a group of four microsatellite markers (RM26, RCS9, RCS5, and RM22) did not amplify in populations T1 and T2, another set of three markers (RM3, RM12, and – again-RM22) failed to amplify in population T3 (Figure 2B). Furthermore, the observed heterozygosity of most markers in populations T1, T2, and T3 is either (very close to) one or (approaching) zero. This indicates the presence of null alleles.

The PCA based on the *R. spiralis* markers (Figure 2D) strongly separates populations T1, T2, T3 and D1b from pure *R. spiralis* along the first axis, which explained 17.6% of the variation. The hybrids can also be placed in two separate groups, following the second axis (explaining 9.4%). The first hybrid cluster contains populations T1, T2, and D1b, the second only population T3. This clustering pattern was confirmed with the DAPC (Figure 2F). We used *K* = 6, based on the lowest BIC, but changing the number of clusters did not alter the pattern of two distinct hybrid clusters. This hybrid clustering pattern corresponds with the previous results where markers were shown to behave differently between populations T1–T2 and population T3. Although the amplification failure pattern (Figure 2A) is partly responsible for this structure, a closer look on the allele frequencies (Supplementary Material S1) reveals that there is very little overlap in alleles between both hybrid clusters for the *R. spiralis* marker set. Population T3 has also very few alleles in common with pure *R. spiralis.*

A similar clustering pattern could be seen in the PCA based on the *R. maritima* markers, although the pattern is less clear (Figure 2C). The pure *R. maritima* samples and the T3 hybrid cluster are separated from each other along the second axis (explains 9.8% of the variation). The T1-T2 hybrid cluster is spread over a larger area, comprising more variation. However, some samples of population T2 can be found in the T3-cluster. This is confirmed in the DAPC based on the *R. maritima* samples (Figure 2D) where some T2-samples are assigned to the T3 cluster. The *R. spiralis* DAPC already showed that these T2 samples might have a mixed origin of both clusters (Figure 2E). This suggests that gene flow between the two hybrid clusters is possible.

Finally, in the analyses based on the *R. maritima* markers, population D1b is grouped together with pure *R. maritima* both in the PCA (Figure 2C) and DAPC (Figure 2E). Increasing K in this DAPC did not set population D1b apart, nor did it increase the number of hybrid clusters. Taking into account that the *R. spiralis* marker-based analyses grouped population D1b in the T1-T2 cluster, we consider this subpopulation as a group of backcrosses between hybrids and *R. maritima*, with the hybrids as pollen donors.

Microsatellites were also used to detect ploidy levels of these hybrid populations. We found three or four alleles for at least one locus in 40% of the hybrids with a *R. spiralis* chloroplast. Therefore, we consider them as tetraploid. However, we never found more than two alleles in population D1b the hybrids with a *R. maritima* chloroplast. This indicates that this lineage might be diploid, but polyploidy might be missed due to the small sample size.

### Population Genetic Structure of *R. maritima* and *R. spiralis*

The five pure *R. maritima* populations were considered as diploid, based on the current allele counts and previous studies. They had limited allelic variation with 1.7 alleles per locus on average (Table 1), low gene diversity (*H*_EXP_ = 0.04) and no observed heterozygosity (*H*_OBS_ = 0.00). This results in a low clonal diversity (*R* = 0.04), with only six different MLGs for all pure *R. maritima* samples (Table 3). Testing for clonal structure with GenoDive could not confirm that the observed clonal structure is truly the result of clonal growth (*p* > 0.05) and all samples were kept in the dataset for further analyses. Based on other parameters, these MLGs are rather the result of strongly inbred lineages: seven loci were monomorphic (RMV56, RMB55, RMB15, RMB53, RMV10, RMB12, and RMB16) and hence removed for further species-specific analyses (Table 2). A complete absence of heterozygosity maximized the inbreeding coefficient (*G*_IS_ = 1). As shown in Table 2, population differentiation parameters G_ST_ and ρ were very high (respectively, 0.80 and 0.82). These parameters are based both on observed and expected heterozygosity, hence the absence of observed heterozygosity in all samples will give a distorted image. Allelic differentiation (*D*_EST_ = 0.41), based on the number of effective alleles, was less biased than G_ST_ and ρ but is affected by the low number of polymorphic loci. The PCA and DAPC based on the *R. maritima* marker set confirmed that there is no strong population structure in this pure *R. maritima* dataset (Figure 2C).

**TABLE 3:** Distribution of the genotypes of pure *R. maritima* among the five pure *R. maritima* populations.

	Genotype 1	Genotype 2	Genotype 3	Genotype 4	Genotype 5	Genotype 6
D1	0%	88%	12%	0%	0%	0%
D2	0%	0%	0%	0%	100%	0%
D3	12%	0%	0%	0%	88%	0%
D4	0%	0%	0%	13%	67%	20%
D5	0%	100%	0%	0%	0%	0%
Total	3%	41%	2%	2%	49%	3%

The twelve pure *R. spiralis* were considered as tetraploids because we found three or four alleles in 60% of these samples. The populations had an overall high clonal richness (Table 1). Only two populations showed substantial clonal growth (P5 with *R* = 0.08 and P7 with *R* = 0.67). Testing for clonal structure revealed that these MLGs are truly the result of clonal growth (*p* < 0.05) and hence only one sample of each MLG is kept. Three populations had one or two MLGs that occurred twice (D6 with *R* = 0.97, D7 with *R* = 0.96 and P8 with *R* = 0.94). We could not confirm that these MLGs were true clones (*p* > 0.05) and all samples are kept. The *R. spiralis* populations had 11.8 alleles per locus on average, but the mean number of effective alleles is 2.5, thereby indicating low evenness (Table 2). The overall heterozygosity (*H*_OBS_ = 0.66 and *H*_EXP_ = 0.59) was comparable but there were large differences among loci, possibly partly as a result of tetraploidy. Overall G_IS_ for *R. spiralis* was negative (−0.18). We found little population differentiation (*G*_ST_ = 0.11, ρ = 0.08, and *D*_EST_ = 0.21), which is concordant with the observed pattern in both the PCA and DAPC (Figure 2). Within the DAPC, the pure *R. spiralis* samples have a scattered distribution over the four remaining clusters (*K* = 6 but two clusters belong to the hybrids), without a clear population structure. Changing the value of *K* did not affect the pattern of hybrid clustering.

### Population Structure of the *Ruppia* Hybrids

The three inferred hybrid populations had a genetic diversity that was intermediate to their parental species (mean number of alleles per locus = 5.0, Table 2). The *R. spiralis* multiplex detected 40 alleles in the hybrid samples, of which ten were unique for the hybrids. Six of them were found exclusively in T3, sometimes strongly differing in allele lengths from the pure *R. spiralis* populations. Besides, there was also little overlap in alleles between the T1-T2 cluster and the T3 cluster. The *R. maritima* multiplex detected 64 different alleles in these hybrid samples, whereas only 17 alleles were found in pure *R. maritima.* Except for one unique allele, all *R. maritima* alleles were re-encountered in the hybrid populations. Contrary to the *R. spiralis* marker set, population T3 has the lowest number of alleles per locus of these hybrid populations and only five unique alleles. Population T1 has the largest number of *R. maritima* alleles (mean allele per locus = 5.7, Table 1).

We also observed strong differences between the two sets of markers in terms of clonality (Table 1). The *R. maritima* marker set found low clonality in T2 (*R* = 0.87) and T3 (*R* = 0.5) but clonal structure could not be confirmed for T3 (*p* > 0.05), probably due to the low number of alleles. Adding the *R. spiralis* markers, no clones could be detected anymore in T2. Overall, clonal richness was estimated at 0.89 with both sets of microsatellites. Considering gene diversity, we found especially large differences among the *R. spiralis* markers: three loci (RM12, RCS9, and RM27) were fixed heterozygotes for one or more hybrid populations, other loci were completely monomorphic (RM3, RM2, and RMB53) (Table 2). The observed and expected heterozygosity was more balanced for loci of the *R. maritima* multiplex. Overall, we found higher population differentiation among the hybrid lineages compared to the pure species.

### Associations Between Habitat and Genetic Lineages

*Ruppia maritima* was found exclusively in temporary ditches, the hybrids exclusively in the shallow temporary ponds and *R. spiralis* was most often encountered in large semi-permanent ponds (Figure 1C). The Fisher test confirmed that these associations between each lineage and habitat type exist (*p* < 0.05 for each contingency table).

## Discussion

In this paper, we used genetic tools to identify different lineages of the genus *Ruppia* that co-occur within a single Mediterranean wetland. We particularly focused on the presence of hybrid lineages. In addition, we combined both genetic and ecological data to detect differences in ecology and habitat use between these different *Ruppia* lineages. Our results show that both *R. spiralis* and *R. maritima* can co-occur within the same wetland area but have different reproductive ecologies and habitat preferences. We also detected several populations of later-generation hybrids. These hybrid populations occupied a distinct habitat, different from the habitat of the parental species. We could not detect any signs of recent hybridization events (F1 hybrids) or frequent introgression between *R. spiralis* and *R. maritima*, although we detected one recently backcrossed lineage, presumably between a local hybrid population and *R. maritima.*

### Different *Ruppia* Lineages Have Different Habitat Preferences

We found strong differences in reproductive strategy and habitat preference between *R. spiralis, R. maritima* and the hybrids. *R. spiralis* was mainly found in large semi-permanent ponds but also occasionally detected in the two temporary habitat types. Previous molecular and detailed ecological studies already revealed that *R. spiralis* has a strong preference for more permanent waterbodies, including some marine environments (Verhoeven, 1979; Mannino and Graziano, 2014; Triest and Sierens, 2014; Martínez-Garrido et al., 2017; Triest et al., 2018). However, several of the *R. spiralis* ponds detected in this study dry out regularly during late summer. This indicates that *R. spiralis* can occupy a large range of habitats in terms of hydroperiod. Nevertheless, we consider these semi-temporary ponds as the edge of their habitat range because droughts too early in summer will prevent *R. spiralis* to complete its life cycle and produce seeds. Besides, occasional summer droughts also prevent the establishment of long-living perennial *R. spiralis* populations as were previously described in more permanent waterbodies (Gesti et al., 2005; Mannino et al., 2015). Previously, a link between clonal growth and habitat stability was suggested (Martínez-Garrido et al., 2017) but our results could not support this hypothesis. Almost all populations showed high levels of outcrossing and low levels of clonality, even the adult populations without flowers, possibly because none of the included ponds provides sufficient stability. The only highly clonal population, population P5, was found in an abandoned salt pan that can exhibit strong fluctuations in water level and is hence not an ecologically stable environment.

Both *R. maritima* and the hybrid populations had a strong preference for temporary waterbodies that dry out during late spring. Several authors already mentioned the preference of European *R. maritima* for more temporary habitat types (Verhoeven, 1979; Triest and Sierens, 2015) which is in accordance with their ecology: a short life cycle combined with high seed set are well-known adaptations of aquatic plants to temporary environments (Brock and Lane, 1983). But despite this known preference for temporary waterbodies, we were surprised to detect *R. maritima* only in a single very specific type of temporary waterbodies: man-made ditches with steep walls. These ditches are regulated by sluices and can undergo sudden and unexpected changes in salinity and water-level, which could impose severe stress on plants (Howard and Mendelssohn, 1999). The shallow temporary ponds, often found in small depressions in the dunes or marshes, dry out more gradually and are hence considered as a less stressful environment. However, these ponds were inhabited by *Ruppia* hybrid populations and a single *R. spiralis* population. This very narrow habitat range of *R. maritima* could possibly be explained by its dominantly selfing reproductive strategy. In selfing species, niche breadth is known to decrease rapidly over time, and selfing species occupy a smaller and more marginal niche compared to their outcrossing relatives (Totté et al., 2015; Park et al., 2018), which is consistent with the narrow habitat range observed in this study. Based on *R.* maritima flower morphology, Verhoeven (1979) already suggested that *R. maritima* is a dominantly selfing species, and this was confirmed with microsatellites by Triest and Sierens (2015). However, these authors found at least some levels of heterozygosity in most European *R. maritima* populations, whereas our populations were completely homozygous with very low levels of genetic diversity. Possibly, low levels of outcrossing are masked by this low genetic diversity and due to the low level of genetic variation, we cannot distinguish between sexual and asexual reproduction, but overall, we can conclude that the *R. maritima* populations in this study are highly selfing.

Established hybrid lineages are often found in different habitats than their parental species (Brochmann et al., 2004; Soltis et al., 2004; Abbott et al., 2013). Hybridization leads to new phenotypes and some newly formed hybrids can survive in habitats that were unfavorable to their parents. The presence of a large variety of niches close to the parental contact zone increases the chances that a new phenotype can colonize a suitable niche (Arnold, 1997). However, most established hybrid lineages are often found in environments that are more extreme in terms of environmental stress or requirements, compared to their parental niches (Burke and Arnold, 2001; Rieseberg et al., 2007; Gramlich et al., 2016). Within our study area, the *Ruppia* hybrids are found in the shallow temporary ponds which is a more intermediate habitat in terms of environmental stress compared to their parental habitats. It is possible that these temporary ponds were an empty gap in the *Ruppia* niche range. The newly formed *Ruppia* hybrids may have been able to colonize these shallow temporary ponds because of the absence of competitors, followed by local adaptation in the following generations (Burke and Arnold, 2001). However, hybridization can also be a mechanism to get rid of possible deleterious genetic “load,” especially in genetically impoverished populations (Ellstrand and Schierenbeck, 2000). Consequently, hybrids can be more fit than their genetically poor progenitor species in their native range (Barton, 2001; Burke and Arnold, 2001). It could be hypothesized that strongly homozygous and genetically poor *R. maritima* was originally found in these shallow temporary ponds as well but had a lower fitness compared to the newly formed hybrids that invaded these habitats. Therefore, the hybrids that colonized these temporary ponds were able to locally outcompete *R. maritima*. Strong selection and local adaptation could not only decrease the niche breadth of a selfing species but also cause a niche shift (Levin, 2010), two mechanisms that might have banned *R. maritima* to the temporary ditches. Although hybrids are generally thought to be less fit than their parental species, especially in their parent’s niche, several examples contradict this (Arnold et al., 2012). For instance, F1 hybrids in North American cattail were found to dominate their parents’ habitats (Olson et al., 2009; Zapfe and Freeland, 2015) and later-generation hybrids between two wild species of the genus *Vigna* are found to be more vigorous in one parent’s native habitat (Takahashi et al., 2015). However, comparisons with other *Ruppia* hybrid zones are necessary to validate this hypothesis, as well as more detailed descriptions of *R. spiralis* and *R. maritima* niches. In the long run, the increased genetic diversity in *Ruppia* hybrids can lead to an increased evolutionary potential which may give these hybrids a competitive advantage compared to the strongly homozygous parental *R. maritima*.

### Origin of the Hybrid Populations

The three hybrid populations can be divided into two different genetic clusters. The first hybrid cluster comprises populations T1 and T2, the second population T3. Each cluster is characterized by a specific group of non-amplifying loci and a set of unique alleles. However, this does not mean that each cluster only groups the interbreeding offspring of a single hybridization event. If a hybrid lineage has a single origin, all hybrid alleles that were not present in the parental individuals would be the result of mutations (Soltis and Soltis, 1993). The genetic diversity in both hybrid clusters probably results from several hybridization events and backcrossing events with mutually outcrossing offspring, supplemented with new alleles due to mutations. Consequently, we assume that both clusters had multiple origins (Meimberg et al., 2009).

Comparing alleles between hybrids and parental species can reveal information about the origin of these hybrids. The *R. spiralis* markers suggested that both hybrid clusters had very little overlap in alleles. The alleles in the T1-T2 cluster largely correspond to those in the local *R. spiralis* populations. However, the T3 cluster had many unique alleles that strongly differ from the current local *R. spiralis* populations. As a result, we assume a local origin for the T1-T2 cluster, whereas the T3-cluster might have originated outside of the Camargue.

Several *R. spiralis* markers completely failed to amplify in one of the hybrid clusters. During the first generations after a hybrid lineage, severe chromosomal rearrangements can take place in a hybrid’s genome including the deletion, inversion and translocation of chromosomal segments, until the chromosomes are stabilized (Adams and Wendel, 2005; Fontdevila, 2005; Baack and Rieseberg, 2007). Studies of polyploidization events in grasses found that up to 15% of the nuclear DNA can be eliminated already during the formation of an F1 hybrid but always from the same parental genome (Levy and Feldman, 2002). If these chromosomal rearrangements or new mutations affect the loci of the included microsatellites, or the primer binding sites, this might result in amplification failure. These chromosomal rearrangements are suggested as a mechanism that could increase reproductive isolation between the hybrid and one or both of the parental species (Schumer et al., 2015).

The *R. maritima* markers support the division into these two hybrid clusters, both hybrid clusters had several alleles that were not detected in the local *R. maritima* populations. These alleles might have accumulated over time through mutation in the hybrid lineage. However, it is also possible that they are the result of several backcrosses between these hybrid lineages and pure *R. maritima* populations in the past. The *R. maritima* populations included in this study were genetically poor, contrary to the hybrid populations. Selfing is a mechanism that reduces genetic diversity rather fast and selfing organisms are prone to extinction-recolonization cycles (Levin, 2010). As a result, it is possible that earlier local *R. maritima* populations contained different alleles that have disappeared over time due to extinction events or remained present in the seed bank (Vandvik et al., 2016). Triest and Sierens (2015) detected one pure *R. maritima* population in the Camargue, but this population had more alleles than the current five populations together. Although the same location did not harbor a *R. maritima* population during our field survey, it is possible that these alleles are still present in the seed bank or in local populations that were not detected during our sampling. Therefore, back-crossings between the hybrids and *R. maritima* at different moments in time might accumulate different *R. maritima* alleles in the hybrid lineages. Followed by outcrossing, these alleles are less prone to extinction in hybrid populations, compared to *R. maritima* populations (Todesco et al., 2016).

Although the majority of angiosperm hybrid lineages has multiple origins, outcrossing between hybrid lineages with different origins is not ubiquitous in the plant kingdom. Within the macrophyte genus *Potamogeton* for instance, many long-living hybrids are known, but the majority of these hybrids are sterile and only reproduce through clonal growth (Kaplan and Fehrer, 2006; Kaplan et al., 2009, 2018). The Asteraceae genus *Tragopogon* has two well-known and recurrently formed hybrid species, *T. mirus* and *T. miscellatus* that can reproduce sexual, but mainly through selfing. Outcrossing between different genotypes has been detected, but only between genotypes that are very similar (Symonds et al., 2010). However, within our study, the absence of high levels of clonality and inbreeding within each cluster suggests that these *Ruppia* hybrids can reproduce sexually with each other. The indications of recent gene flow between these two clusters even hints that outcrossing between hybrids with a different genetic history is possible, despite their genomic differences. Outcrossing between hybrids that result from different hybrid crosses can strongly increase the genetic diversity of a hybrid lineage with multiple origins. This could promote the adaptive potential of these hybrids, and hence increase the potential for hybrid speciation (Meimberg et al., 2009).

We could not detect F1 hybrids, nor were they detected in previous molecular studies (Triest and Sierens, 2015; Martínez-Garrido et al., 2016). It is possible that prezygotic reproductive barriers such as differences in ploidy or mating system prevent frequent hybridization between the tetraploid outcrossing *R. spiralis* and the diploid selfing *R. maritima.* Both differences in ploidy and mating system are considered as important reproductive barriers that promote speciation, although they are also known to be permeable to some extent (Brandvain and Haig, 2005; Goodwillie and Ness, 2013; Hülber et al., 2015). For instance, within the well-studied genus *Mimulus* several examples are known of species with different mating systems or ploidy that still hybridize. The predominantly selfing *M. nasutus* frequently hybridizes with its outcrossing relative *M. guttatus* (Brandvain et al., 2014). *M. guttatus* is also a good example of how a ploidy barrier can be permeable: this diploid species was introduced in the United Kingdom in 1812, where it hybridized with tetraploid *M. luteus*. The offspring, the sterile triploid hybrid species *M. x robertsii* has multiple independent origins and is widespread throughout the United Kingdom (Vallejo-Marín and Lye, 2013). However, somehow *M. x robertsii* has overcome this hybrid sterility which resulted in the recently discovered fertile allopolyploid species *M. peregrinus* (Vallejo-Marín et al., 2015). Although triploids are dominantly sterile, the rare production of unreduced gametes that can cross with each other or backcross with one of the parental species, assures that a hybrid lineage can persist. This mechanism is also suggested in the genus *Senecio* where introgression from a diploid species into a tetraploid species results in a phenotype with flowers that are more attractive to pollinators (Chapman and Abbott, 2010). Some authors consider ecological factors such as differences in flowering time or pollination mode as even more important reproductive barriers than differences in ploidy or mating system (Brandvain et al., 2014; Briscoe Runquist et al., 2014): *R. maritima* flowers during spring whereas *R. spiralis* flowers during summer, when most *R. maritima* ponds have already dried up. As a result, successful fertilization between these two species is unlikely. If there is an overlap in flowering time and cross-fertilization can take place, a fertilized *R. spiralis* flower in a more permanent waterbody is more likely to produce a mature seed compared to a fertilized *R. maritima* flower in an almost dried-up pond. This might explain why no hybrids with a *R. maritima* chloroplast were detected so far (Triest and Sierens, 2015; Martínez-Garrido et al., 2016). Furthermore, although F1 hybrid formation is rare, this does not necessarily prevent the formation of well-established later-generation hybrid lineages. In the genus *Iris*, F1 hybrids between the two Louisiana species *I. fulva* and *I. hexagona* are rare. Nevertheless, the presence of well-established later-generation hybrid populations as well as high levels of adaptive introgression from one species into the other indicates that even rare hybridization might result in persistent hybrid lineages. Arnold (1997) suggests that once hybridization can overcome the reproductive barriers and F1 hybrids are produced, further hybridization is accelerated.

We detected fourteen samples that turned out to be backcrossings between *R. maritima* and the local hybrids. These samples are placed in subpopulation D1b that is found mixed with the pure *R. maritima* samples of population D1. They are the first described *Ruppia* hybrids with a *R. maritima* chloroplast. The reproductive barriers between *R. maritima* and the tetraploid outcrossing hybrids are similar to those between *R. maritima* and *R. spiralis*, which indicates that differences in mating system or ploidy are somewhat permeable barriers in this genus. However, both *R. maritima* and the hybrid populations were found in ponds that dry out during late spring. An overlap in flowering time between these two lineages seems hence more likely compared to *R. maritima* and *R. spiralis*. This would increase the chances of *R. maritima* as the maternal parent of a backcross. Followed by selfing, this could result in introgression of hybrid alleles into *R. maritima*. In selfing species, introgression could potentially be considered as a mechanism to rescue them from a build-up of deleterious alleles (Brandvain et al., 2014) or replace damaged alleles (Rieseberg, 2009). Although we could not detect any signs of frequent introgression in *R. maritima*, even low levels of introgression might be sufficient to maintain adequate levels of genetic diversity in this species. Triest and Sierens (2015) detected two populations with a *R. spiralis* chloroplast that only had a *R. maritima* ITS-region, which suggests strong introgression of *R. spiralis* toward *R. maritima*, potentially combined with ongoing *R. spiralis* chloroplast capture. These populations exhibited a higher genetic diversity and a lower inbreeding depression compared to pure *R. maritima*. On a large scale, the presence of *Ruppia* hybrids might act as a genetic reservoir for the genetically poor *R. maritima*, that could prevent this selfing species from its direct way to extinction (Todesco et al., 2016).

One limitation in our study is the absence of a good set of cross-amplifying markers that amplified in all samples. In the design of our multiplexes, we tried to overcome this problem by including markers RMB5, RMB15, and RMB53 in both multiplexes, but we did not foresee that only RMB15 could amplify successful in all populations. We also added diagnostic markers RUMR4 and RM3 to the *R. spiralis* multiplex but we omitted them from the *R. maritima* multiplex because they could not detect variation within pure *R. maritima* (Triest et al., 2017). Considering the structure of our data, we believe that our methods allowed us to detect a wide range of hybrids. For instance, a hybrid with a *R. maritima* chloroplast, would have at least some traces of heterozygosity or allele deviations from pure *R. maritima*. An F1-hybrid that was only amplified with the *R. spiralis* multiplex, would have different alleles for diagnostic markers RM3 and RUMR4. However, for good practice, we would advise to always carefully consider the composition of the multiplexes for the detection of hybrids and introgression. We strongly advice to include RM3 and RUMR4 in the *R. maritima* multiplex. We would also add some very specific markers of the other species in each species-specific multiplex, which would allow to detect extra alleles, that directly descend from hybridizations.

Flower peduncle length is often considered as the clearest distinguishing characteristic between *R. spiralis* and *R. maritima*. However, all populations that turned out to be *Ruppia* hybrids or pure *R. maritima* had fully grown flowers at the moment of sampling, all with short peduncles (<5 cm). Subsequently, we considered these hybrid populations as *R. maritima* during sampling. Previous reports of *Ruppia* hybrids also noticed that they wrongly identified *Ruppia* hybrids as *Ruppia maritima* in the field (Triest and Sierens, 2013, 2015; Martínez-Garrido et al., 2016). Verhoeven (1975) described the typical habitat of *R. maritima* in the Camargue as both temporary ditches and ponds, hence he might have possibly identified hybrids as *R. maritima.* This apparently common misidentification of hybrids as *R. maritima* probably partly contributes to the overestimation of the range and local abundance of *R. maritima* (Triest and Sierens, 2015). Therefore, we strongly recommend the use of genetic markers to identify different *Ruppia* species. Other studies on aquatic plants report a similar trend with a complex taxonomy within a genus that is resulting from cryptic species and hybridization events. Within the genus *Najas* for instance, *N. marina*, *N. major*- which was previously considered as subspecies of *N. marina*- and their subsequent sterile F1 hybrids can only be identified using molecular markers (Triest, 1988; Rüegg et al., 2019). The wide-ranging taxon *N. flexilis* turned out to be two morphologically similar but genetically distinct species (Les et al., 2015). In *Potamogeton*, a genus where hybridization is well described and recognized since the 18th century, several North American F1 hybrid species that were detected with molecular markers, turned out to be overlooked by previous morphological studies (Kaplan et al., 2009). Identification within the aquatic *Ranunculus* subgenus *Batrachium* has always been difficult and only recently, molecular studies revealed that the morphological highly diverse species complex *R. penicillatus* is probably rather a hybrid swarm than a distinct species group or a diverged hybrid lineage (Zalewska-Gałosz et al., 2015). This cryptic diversity that is observed in many macrophyte taxa leads to an underestimation of biodiversity. Molecular markers and a better understanding of hybridization events are therefore crucial to understand the true biodiversity of inland aquatic systems.

## Conclusion

The Camargue harbors both *R. spiralis* and *R. maritima* as well as several lineages of a hybrid origin. These encountered later-generation hybrid lineages co-occur with their parental species but are found in a different pond type. They are genetically differentiated from both parental species, although back-crossings between hybrids and - at least - *R. maritima* remain possible. Our dataset suggests that these two species and their hybrids have different habitat preferences, different reproductive ecologies and different ploidies. These differences could act as strong prezygotic reproductive barriers, preventing frequent hybridization and introgression, but are permeable to some extent. So far, these hybrids could not be identified based on morphology, which strongly increases the taxonomic complexity in this genus. However, many aquatic plants are morphologically cryptic, and it is not unlikely that, as in *Ruppia*, closer examination of their genetic composition would reveal similar complex structures of coexisting hybrids, backcrosses and parental species. Given that similar complexity has been found in the hybrid complexes of freshwater zooplankton such as Daphnia (Thielsch et al., 2017), such patterns of cryptic coexistence of hybrids and parental species may be even more common in freshwater environments than previously assumed.

## Data Availability Statement

The microsatellite data for both species are available at Dryad (doi: 10.5061/dryad.sn02v6x1c).

## Author Contributions

LB has performed the fieldwork and laboratory work, analyzed the data, and wrote the manuscript. LT helped during the field work and contributed laboratory equipment. LT and BV helped during data-analysis and manuscript writing. All authors contributed to the manuscript and approved the submitted version.

## Conflict of Interest

The authors declare that the research was conducted in the absence of any commercial or financial relationships that could be construed as a potential conflict of interest.

## Supplementary Material

MATERIAL S1 Allele frequencies of each microsatellite, calculated for each population.
